# 3D Pd/Co core–shell nanoneedle arrays as a high-performance cathode catalyst layer for AAEMFCs†

**DOI:** 10.1039/c7ra13677c

**Published:** 2018-04-05

**Authors:** Jia Jia, Hongmei Yu, Xueqiang Gao, Jun Chi, Yachao Zeng, Bowen Qin, Dewei Yao, Wei Song, Zhigang Shao, Baolian Yi

**Affiliations:** Fuel Cell System and Engineering Group, Dalian Institute of Chemical Physics, Chinese Academy of Sciences 457 Zhongshan Road 116023 Dalian P. R. China hmyu@dicp.ac.cn zhgshao@dicp.ac.cn +86 411 84379185 +86 411 84379051; University of Chinese Academy of Sciences 19A Yuquan Road 100049 Beijing P. R. China

## Abstract

A novel cathode architecture using vertically aligned Co nanoneedle arrays as an ordered support for application in alkaline anion-exchange membrane fuel cells (AAEMFCs) has been developed. The Co nanoneedle arrays were directly grown on a stainless steel sheet *via* a hydrothermal reaction and then a Pd layer was deposited on the surface of the Co nanoneedle arrays using a vacuum sputter-deposition method to form Pd/Co nanoneedle arrays. After transferring the Pd/Co nanoneedle arrays to an AAEM, a cathode catalyst layer was formed. Without the use of an alkaline ionomer, the AAEMFC with the prepared cathode catalyst layer showed an enhanced performance with ultra-low Pd loading of down to 33.5 μg cm^−2^, which is much higher than the conventionally used cathode electrode with a Pt loading of 100 μg cm^−2^. This is the first report where three-dimensional Co nanoneedle arrays have been used as the cathode support in an AAEMFC, which is able to deliver a higher power density without an alkaline ionomer than that of conventional membrane electrode assembly (MEA).

## Abstract 2

A novel ordered Pd/Co nanoneedle array was used as a cathode in an AAEMFC and delivered a higher power density than that of conventional MEA.

## Graphical abstract

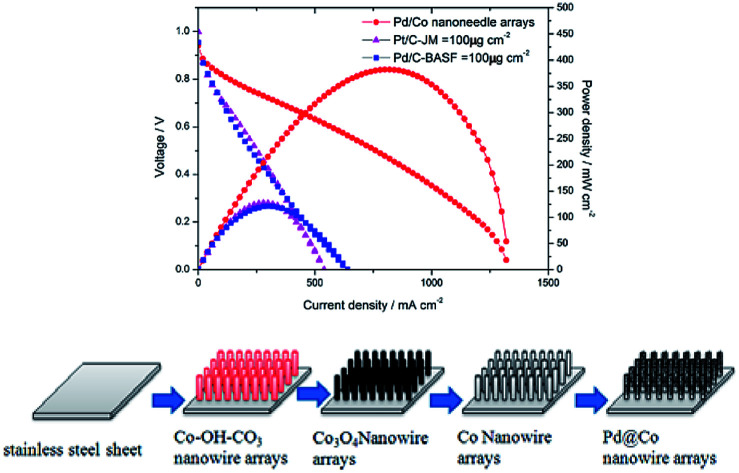

## Introduction

Over the past few years, a new class of fuel cells, alkaline anion-exchange membrane fuel cells (AAEMFCs), has gained growing interest in the research community as a lower cost alternative to proton exchange membrane fuel cells (PEMFCs).^1–3^ Technically, the main difference between AAEMFCs and PEMFCs is that AAEMFCs use an alkaline anion-exchange membrane (AAEM) as the solid membrane rather than the acidic membrane used in PEMFCs. The use of an AAEM creates an alkaline environment in the cell and therefore AAEMFCs have some outstanding advantages over PEMFCs: (a) they have faster electrochemical reaction kinetics for the oxygen reduction reaction (ORR); (b) minimized corrosion issues, allowing for the use of less expensive, non-Pt or non-precious metal catalysts; (c) lower fuel crossover rates; (d) an extensive range of materials can be used in the cell and stack components.

In recent years, the data of higher performance AAEMFCs have been reported, with the peak power density surpassing 1 W cm^−2^. This performance improvement is mainly due to the outstanding progress of anion-exchange membranes (AEMs) with higher anion conductivity.^4–6^ However, little attention has been paid to the architecture of the catalyst layers (CLs) since it is crucial to the performance of AAEMFCs. State-of-the-art methods for the preparation of CLs for AAEMFCs, taken from the methods used to synthesize its acidic counterpart PEMFCs, include the preparation of a catalyst coated membrane (CCM) where the CLs are formed by directly coating a slurry of a catalyst on both sides of the AEM and the gas diffusion electrode (GDE), where the CL is brushed on the microporous layer side of the gas diffusion layer.^7–9^ The CLs are composed of finely divided particles of a platinum group metal (PGM) supported on carbon and mixed with an ionomer (an ion conducting polymer). This structure provides conductive pathways for electrons through the carbon support, hydroxyl ions through the ionomer, and gas diffusion pathways for reactants through gas-filled pores.^10,11^ However, these conventional electrode designs may impose constraints on the performance of AAEMFCs, since the ionic conductivity of state-of-the-art anion ionomers is not high enough and the stability is also not good enough. In addition, the random structure of the state-of-the-art catalytic layers comprising a mixture of electrons, ions and gas conducting components will restrict the catalyst utilization efficiency.

To overcome these problems, an effective way is to develop advanced catalyst layers and significant gains could be achieved if the catalyst layer has an optimized spatially organized structure. Recently, three dimensional (3D) nanostructured materials have emerged as catalyst supports in PEMFCs.^12–16^ The nanostructured thin films (NSTFs) developed by Debe *et al.* of the 3M Corporation are composed of arrays of small organic whiskers (of less than 1 μm in length), fully covered with a thin Pt or Pt-based alloy catalyst layer.^12^ The NSTF electrodes exhibit a significant activity improvement over that of a conventional Pt/C-based electrode due to the thin film structure of the catalyst, and the ordered and ultrathin structure of the CLs that can facilitate mass transfer at a high current density. Inspired by the NSTFs of 3M, several groups came up with alternative materials. Tian *et al.* introduced vertically aligned carbon nanotubes (VACNTs) as supports to construct an ordered electrode structure with a high Pt dispersion and low loading.^16^ The Pt/VACNT electrodes also showed a high performance due to the ordered VACNTs providing a more efficient and uniform reaction environment for the Pt catalysts. Kim *et al.* developed an ordered macroporous inverse opal structured (IP) electrode using polystyrene (PS) beads as the template.^15^ The IP electrode shows a promising performance because the structure can offer a relatively large surface area, large voidage, low tortuosity and interconnected macropores, which can effectively improve mass transfer and water management. Besides this example, spatially ordered metal oxide structures, such as vertically aligned TiO_2_ nanotube arrays, have also been investigated as an oriented support for PEMFCs and show excellent long-term electrochemical durability.^14^

Despite these significant advantages, only a few studies have reported the use of these advanced catalyst layers in AAEMFCs.^17,18^ Our previous work using Co–OH–CO_3_ and Cu as ordered supports suggested that the application of an ordered nanostructure in the cathode catalyst layer can significantly improve the catalyst utilization efficiency and the AAEMFC performance, especially the high current density and eliminate the reliance on alkaline ionomers with low hydroxide ion conductivity. However, there are some drawbacks to the use of Co–OH–CO_3_ and Cu as ordered supports. The structure of the developed Pt/Co–OH–CO_3_ nanowire arrays will collapse after acid washing and the cohesion of the AEM to the GDE type of Pd/Cu nanoneedle array electrode needs to be improved in order to reduce interface resistance and gain a better cell performance. In order to solve the problems associated with the use of Co–OH–CO_3_ and Cu as ordered supports, a CCM-type ordered electrode is expected to resolve the issues encountered and acid washing is not essential after transferring the arrays to the AEM, thus avoiding the collapse of the structure. In the meantime, the high cost and scarcity of Pt, which is widely used in catalysis, are the major impediments for the commercialization of fuel cells. The alkaline environment brought about by the presence of an AEM in the fuel cell creates the possibility of replacing Pt with a Pt-free catalyst.^19–24^ However, there are only a few studies where Pt-free catalysts have been used in AAEMFCs as a cathode catalyst. Among them, Pd-based catalysts seem to be the most promising alternative to Pt-based catalysts, with competitive intrinsic electrocatalytic performance towards the ORR compared to that of Pt.^19,24^

In this work, we introduce an advanced CCM-type 3D cathode architecture using novel Co nanoneedle arrays as a catalyst support for AAEMFCs. The Co nanoneedle arrays not only retain the advantages of Cu nanoneedle arrays,^18^ but also overcome the shortage of the weak cohesion between the Cu nanoneedle arrays and the AEM. At the same time, Pd was chosen as the cathode catalyst metal because of its high activity in alkaline media. With its novel electrode structure, the Pd catalyst utilization efficiency can be improved, thus further improving the cell performance.

## Experimental

### Preparation of the materials

Firstly, Co–OH–CO_3_ nanoneedle arrays were directly grown on a stainless steel sheet *via* a hydrothermal method. In a typical synthesis, Co(NO_3_)_2_·6H_2_O (0.932 g), NH_4_F (0.148 g) and urea (0.48 g) were dissolved into 40 mL of distilled water by magnetic stirring for 30 min. Then, the homogeneous solution was transferred into a 120 mL Teflon lined stainless steel autoclave. A piece of clean 304L stainless steel sheet (2.5 cm × 7 cm) was cleaned with ethanol and distilled water in turn, and then immersed into the above solution. The autoclave was sealed and kept at 120 °C for 5 h. After cooling to room temperature, the stainless steel sheet with pink Co–OH–CO_3_ nanoneedle arrays was collected and washed several times with distilled water. Then, the stainless steel sheet with Co–OH–CO_3_ nanoneedle arrays was put into a muffle furnace and heat treated under an air atmosphere at 400 °C for 2 hours to prepare the Co_3_O_4_ nanoneedle arrays. After cooling to room temperature, the stainless steel sheet with black Co_3_O_4_ nanoneedle arrays was placed into a tubular furnace and reduced using a H_2_/Ar atmosphere at 400 °C for 2 hours to prepare Co nanoneedle arrays. After cooling to room temperature, the stainless steel sheet with black Co nanoneedle arrays was collected.

Secondly, the Pd/Co nanoneedle array electrode was prepared *via* a vacuum sputter-deposition method. In a typical synthesis, Pd was sputtered onto the surface of the Co nanoneedle arrays using a vacuum sputter-deposition technique with a palladium target at 120 W and 1.0 bar of Ar, so as to encapsulate them.

### Characterization of the materials

The morphology and microstructure of the products were characterized using a field emission scanning electron microscope (FESEM, JSM-7800F) with an energy dispersive X-ray spectrometer (EDS) and transmission electron microscopy (TEM) (TEM, JEM2010-HR, 120 kV). The phase and composition of the samples were investigated using powder X-ray diffraction (PXRD, Bruker, D8 ADVANCE) with CuKα radiation (*λ* = 1.5418 Å). The metal loadings of the electrodes were measured by inductively coupled plasma atomic emission spectrometry (ICP-AES) on Leeman Plasma-Spec-I equipment.

### *Ex situ* catalytic activity test

First, 4 mg of Pt/C powder (20% Pt, Sigma-Aldrich), or Pd/Co nanoneedles that were shaved from the stainless steel, was dispersed in 20 mL of isopropyl alcohol and 16 μL of a 5% Nafion solution was added. The mixture was immediately ultrasonicated before use. Next, using a microsyringe, 10 μL of the dispersion was cast onto a glass carbon rotating disk electrode (GC-RDE). This GC-RDE was then tested using linear sweep voltammetry (LSV) measurements. The LSV measurements were conducted in a 1.0 M O_2_-saturated KOH solution at a scan rate of 10 mV s^−1^ and a rotation speed of 1600 rpm.

### MEA fabrication and single cell test

The Pt/Co nanoneedle array electrode was hot-pressed onto one side of a home-made alkaline membrane,^25^ and the other side of the membrane was a home-made catalyst coated membrane (CCM, PtRu loading: 0.1 mg cm^−2^). Two pieces of carbon papers (Toray, TGP-H-060) were utilized as the anode and the cathode gas diffusion layer, respectively, which sandwich the electrode above to form a MEA by hot-pressing at 60 °C and 1 MPa for 2 min. The prepared MEAs were assembled into fuel cells with an effective area of 4 cm^2^.

Fuel cell tests were conducted at 60 °C using H_2_/O_2_ (100% relative humidity, RH) with flow rates of 100/200 mL min^−1^ at 0.2 MPa, respectively. The *i*–*V* curves and high-frequency resistance (RHF) of the single cells were measured and recorded using an electric load system (KMF2030, Kikusui Electronics Corp.). The electrochemical impedance of an AAEMFC was tested using a Solartron cell tester. Impedance spectra were recorded by superimposing a 10 mA AC signal on a different current density in galvanostatic mode with frequencies ranging from 100 kHz to 0.1 Hz.

## Results and discussion

**Scheme 1:**
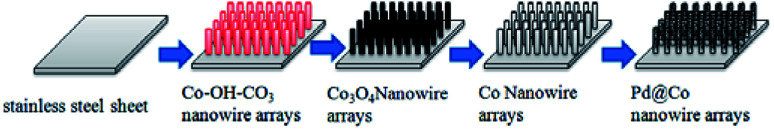
Schematic illustration of the synthesis of a Pd catalyst on Cu nanoneedle arrays.

The fabrication procedure for the 3D nanostructured electrode is schematically illustrated in Scheme 1. A piece of stainless steel sheet was used as a substrate for growing Co–OH–CO_3_ nanoneedle arrays. A solution of Co(NO_3_)_2_, urea and NH_4_F was used as a precursor to prepare Co–OH–CO_3_ nanoneedle arrays on a clean stainless steel sheet under hydrothermal conditions. After the hydrothermal reaction, the stainless steel sheet with the prepared Co–OH–CO_3_ nanoneedle arrays was heated under an air atmosphere to form Co_3_O_4_ nanoneedle arrays on the stainless steel sheet. Then, the stainless steel sheet with the Co_3_O_4_ nanoneedle arrays was then heated and reduced under a H_2_/Ar atmosphere to obtain the Co nanoneedle arrays on the stainless steel sheet. After the reduction reaction, Pd nanoparticles were deposited onto the Co nanoneedle arrays/stainless steel sheet using physical vapor sputtering system. The setup arrangement of the physical vapor sputtering system is shown in Fig. S1.†

**Fig. 1:**
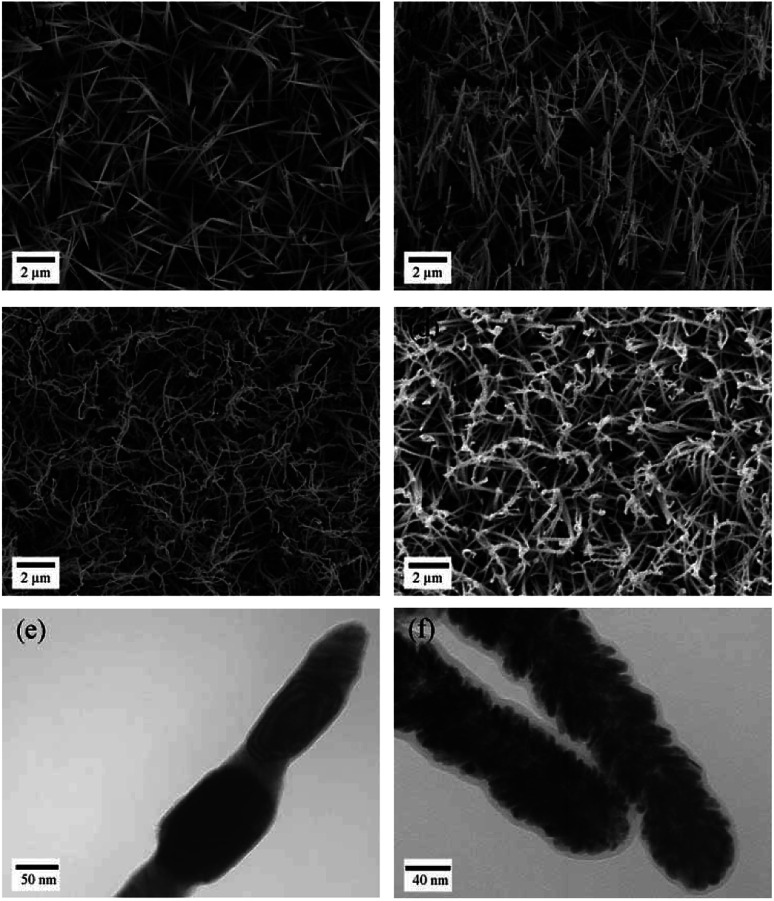
SEM images of (a) Co–OH–CO_3_ nanoneedle arrays, (b) Co_3_O_4_ nanoneedle arrays, (c) Co nanoneedle arrays, and (d) Pd@Co nanoneedle arrays; and TEM images of (e) Co nanoneedle arrays, and (f) Pd@Co nanoneedle arrays.

Typical SEM images of the Co–OH–CO_3_ nanoneedle arrays, Co_3_O_4_ nanoneedle arrays, Co nanoneedle arrays and Pd/Co nanoneedle arrays are shown in Fig. 1a–d, respectively, and typical TEM images of the Co nanoneedle arrays and Pd/Co nanoneedle arrays are shown in Fig. 1e and f, respectively. It can be seen that the Co–OH–CO_3_ and Co_3_O_4_ nanoneedle arrays are homogeneously aligned and grow evenly on the stainless steel sheet (Fig. 1a and b). Both the Co–OH–CO_3_ arrays and Co_3_O_4_ arrays are mainly dominated by needle-like nanostructure arrays with a length of several micrometers and diameter of 80 nm (Fig. 1e). Besides this, the prepared Co–OH–CO_3_ nanoneedles have sharp tips. After a reduction reaction, the morphology of the prepared Co nanoneedle arrays is almost the same as that of the Co–OH–CO_3_ nanoneedle arrays (Fig. 1c). Only the diameters are slightly decreased due to the loss of oxygen in the reduction reaction leading to a shrinking of the volume. After sputtering the Pd nanoparticles onto the surface of the Co nanoneedle arrays, the needle-like nanostructures were maintained (Fig. 1d). However, the sharp tips became dull, which means that the Pd nanoparticles were successfully coated onto the Co nanoneedles. Pd nanoparticles cannot be obviously observed on the surface of the Co nanoneedle arrays in the SEM image, since the diameter of the Pd nanoparticles is only about 5–10 nm and the distribution of the Pd nanoparticles is uniform, done using a DC sputtering technique (Fig. 1f). To further characterize the structure of the Pd/Co nanoneedle arrays, TEM images were taken. A single Co nanoneedle is displayed in Fig. 1e and has a polycrystalline structure morphology similar to that seen in the SEM image. Fig. 1f shows a single Pd/Co nanoneedle and it is very different from the Co nanoneedle in Fig. 1e. It can be clearly observed that the Pd nanoparticles are uniformly coated on the surface of the Co nanoneedle and form a nanostructured thin film structure.

**Fig. 2:**
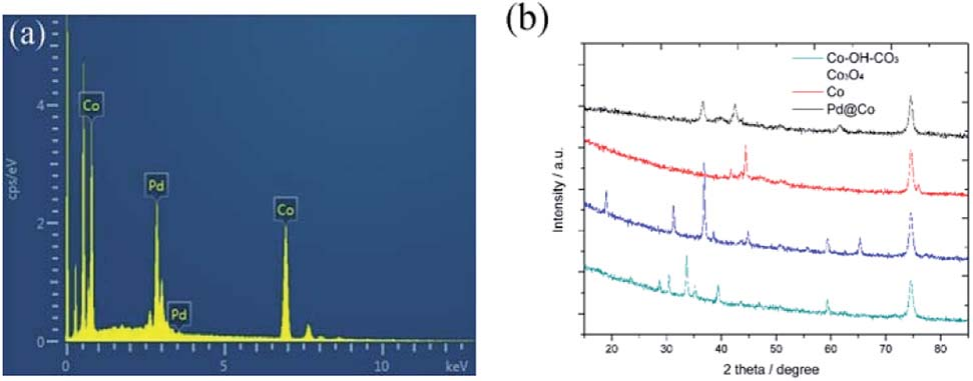
(a) The EDS spectrum of the Pd/Co nanoneedle arrays and (b) the PXRD patterns of the Co–OH–CO_3_, Co_3_O_4_, Co and Pd/Co nanoneedle arrays.

The EDS spectrum and PXRD patterns of the prepared samples are shown in Fig. 2, respectively. Fig. 2a shows the EDS spectrum of the prepared Pd/Co nanoneedle arrays. From the figure it can be seen that the Pd/Co nanoneedle arrays consist of two elements: Pd and Co, the composition ratio of which was found to be 1 : 4 from the EDS results. Fig. 2b shows the PXRD patterns of the Co–OH–CO_3_ nanoneedle arrays, Co_3_O_4_ nanoneedle arrays, Co nanoneedle arrays and Pd/Co nanoneedle arrays, respectively. There are characteristic diffraction peaks of Co–OH–CO_3_ at 28.8°, 30.4°, 33.8° and 35.5° which were assigned to the (121), (300), (221) and (040) faces. The PXRD patterns are consistent with the values on the standard card (JCPDS card no. 048-0083). After heat treating Co–OH–CO_3_ under an air atmosphere, characteristic diffraction peaks at 19°, 31.3°, 36.8° and 38.5° were observed which were assigned to the (111), (220), (311) and (222) faces of Co_3_O_4_ and the PXRD patterns were consistent with the values on the standard card (JCPDS card no. 01-073-1701). Then, after heat treatment of the Co_3_O_4_ under a H_2_/Ar atmosphere, characteristic diffraction peaks at 44.4° and 51.3° were observed which were assigned to the (111) and (200) faces of Co, and the PXRD patterns were consistent with the values on the standard card (JCPDS card no. 01-1259). There are faint peaks at 36.3° and 42.2° that appear after the Pd nanoparticle sputtering, which correspond to the Pd_2_O (111) and (200) crystal faces in the Pd/Co nanoneedle arrays.

**Fig. 3:**
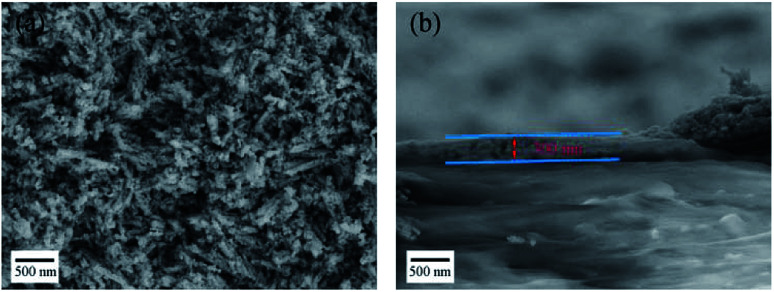
SEM images of (a) the Pd/Co nanoneedle arrays transferred onto the AAEM using a hot press and (b) an image of the cross section of the material after the transfer.

The Pd/Co nanoneedle array coated AAEM was prepared using a hot press. The transfer process does not require any chemical removal of the substrate, and the stainless steel sheet can be easily peeled off from the AAEM without destroying the membrane. The Co nanoneedle arrays grown on the stainless steel sheet can be easily and completely transferred to the AAEM using a hot press due to the weak interaction forces between the Co nanoneedle arrays and the stainless steel sheet. The Pd/Co nanoneedle arrays are uniformly attached to one side of the AAEM and are used as cathode catalyst layer, while the other side of the AAEM is coated with PtRu/C as the anode catalyst layer, done before the transfer process. Fig. 3a shows the conformation of the Pd/Co nanoneedle arrays coated on AAEM after being transferred by hot press, dominated by needle-like nanostructures with a length of less than 1 μm and a diameter of less than 100 nm. Fig. 3b shows a cross sectional image of the Pd/Co nanoneedle arrays coated on the membrane and the thickness of Pd/Co nanoneedle arrays is only about 300 nm, which is 20 times thinner than that of a conventional CCM electrode and 4 times thinner than that of GDE-type Pd/Cu nanoneedle arrays.^18^

**Fig. 4:**
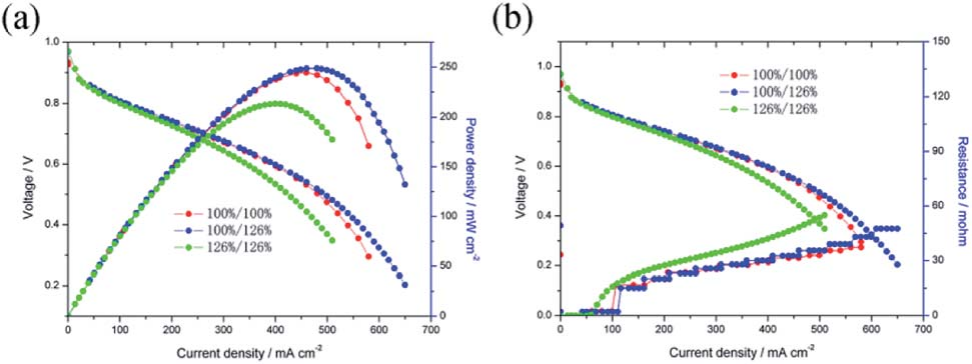
(a) Effect of the inlet gas humidity on the single AAEMFC performance, (b) effect of the inlet gas humidity on the single AAEMFC ohmic resistance.

The catalytic activities of the Pd/Co nanoneedle arrays as the cathode in a single AAEMFC were evaluated using H_2_ and O_2_ at 60 °C, and the *i*–*V* curves and power density are shown in Fig. 4. As found in our previous work, there is no alkaline ionomer to conduct OH^−^ in the novel ordered catalyst layer and water to conduct OH^−^ in the novel cathode catalyst layer.^17,18^ Meanwhile, unlike PEMFCs, the oxygen reduction reaction (ORR) in the cathode of the AAEMFC consumes water which leads to the cathode drying out easily. Therefore, increasing the humidity of the cathode inlet gas is a feasible way of adjusting the water content in the cathode catalyst layer. Fig. 4a and b show the effect of cathode inlet gas humidity on the single cell performance and the ohmic resistance, respectively. The Pd loading of the cathode side of the cell is 33.5 μg cm^−2^. The cathode inlet gas humidity was adjusted by controlling the temperature of a bubbling humidifier. The cell with the higher cathode inlet gas humidity (126% RH, 65 °C in the bubbling humidifier) displayed a higher power density and limiting current density compared to that of the cell with a lower cathode inlet gas humidity (100% RH, 60 °C in the bubbling humidifier). The difference in the performance of the cells with different cathode inlet gas humidity suggests that water plays an important role in conducting OH^−^ in the cathode catalyst layer with the Pd/Co nanoneedle arrays. However, both the anode and cathode with a higher inlet gas humidity (anode 126% RH and cathode 126% RH) show a lower power density and limiting current density compared to a cell with full inlet gas humidity (anode 100% RH and cathode 100% RH). Despite better OH^−^ transport expected as a result of the high inlet gas humidity, the mass transfer polarization loss impacts the cell significantly, resulting in the catalyst layer flooding and hindering the performance of the cell.

**Fig. 5:**
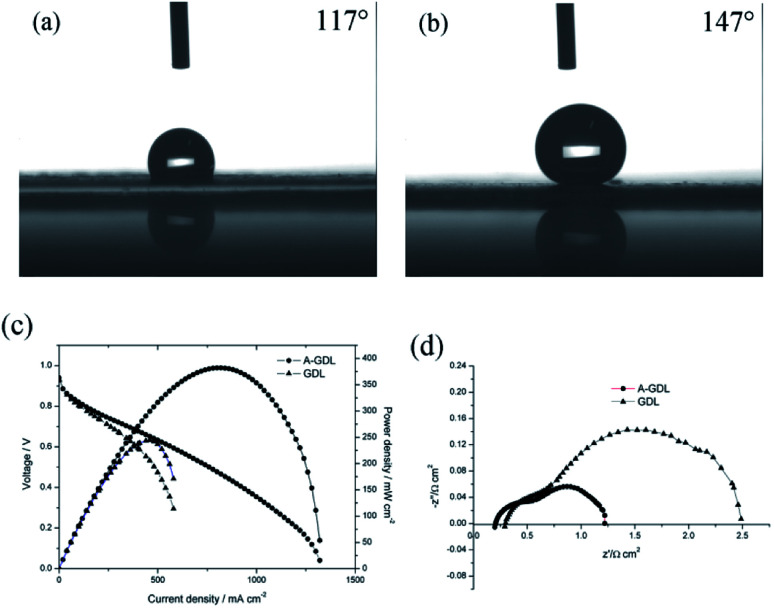
The contact angle images of the different GDLs: (a) the GDL (Sunrise Power) coating with 0.1 mg cm^−2^ of XC-72 carbon, (b) the GDL (Sunrise Power); (c) the *i*–*V* curves and power density of single AAEMFCs using Pd/Co nanoneedle arrays as a cathode; (d) EIS data comparison of AAEMFCs with different GDLs.

Besides this, another way to manipulate the water content in the cathode catalyst layer is to optimize the gas diffusion layer (GDL) of the cathode side.^17^ Due to the Pd/Co nanoneedle array catalyst layer being bound to the AEM, increasing the hydrophilicity of the GDL is also a feasible choice. In our previous work, naked Toray carbon papers (TGP-H-060) were used instead of Toray carbon papers coated with micro porous layer (MPL) in order to decrease the hydrophobicity of the GDL and improve the performance of the AAEMFC.^17^ This time, to further decrease the hydrophobicity of the GDL, 0.1 mg cm^−2^ of XC-72 carbon treated with HNO_3_ was coated on the GDL (Sunrise Power, the Toray carbon papers (TGP-H-060) coated with MPL) surface to enhance the hydrophilicity of the GDL, and named A-GDL. Fig. 5a and b show the contact angle results of A-GDL and the GDL (Sunrise Power), respectively. The A-GDL has a contact angle of 117°, which shows its lower hydrophobicity compared to that of the GDL (Sunrise Power), which has a contact angle of 147°. This means that coating the GDL (Sunrise Power) with 0.1 mg cm^−2^ of XC-72 carbon can significantly increase the hydrophilicity of the GDL. The *i*–*V* curves and power densities of the AAEMFCs with different cathode GDLs are compared in Fig. 5c. The AAEMFC using A-GDL as the cathode GDL displays a significantly higher performance compared with that of the AAEMFC using the GDL (Sunrise Power) as the cathode GDL. In the activation-control region, from an open circuit voltage to 0.85 V, the two cells show almost the same performance. Nevertheless, as the cell voltage decreases to less than 0.85 V (the ohmic resistance and mass transport-control region), the cell performance becomes significantly different. In the ohmic resistance control region, the current density of the cell using the GDL (Sunrise Power) at around 0.6 V is 400 mA cm^−2^ and increases to 580 mA cm^−2^ for the cell using the A-GDL. With an increase in the current density, there is mass transport resistance in the cell using the GDL (Sunrise Power), whereas there is little mass transport resistance in the cell using the A-GDL. The ORR in alkaline media needs water as a reactor and the less hydrophobic A-GDL can provide more water to the catalyst layer compared with the GDL (Sunrise Power), so with an increase in the current density, more water is needed and the A-GDL can provide enough water to reduce the mass transport resistance. By using the A-GDL, the cell shows a maximum current density of 1320 mA cm^−2^, which is 220% higher than that of the cell using the GDL (Sunrise Power) (less than 600 mA cm^−2^). The maximum power density also increases significantly from 244 mW cm^−2^ in the cell using the GDL (Sunrise Power) to 381 mW cm^−2^ in the cell using the A-GDL. The significant difference in the performance of the cells with different cathode GDLs suggests that water plays an important role in conducting OH^−^ in the cathode catalyst layer with Pt/Co nanoneedle arrays. By increasing the hydrophilicity of the GDL, the ohmic resistance and the mass transport resistance of the cell reduce significantly, which means that increasing the hydrophilicity of the cathode GDL is a better way to improve the performance of the AAEMFC compared with increasing the humidity of the cathode inlet gas. The electrochemical impedance data at 300 mA cm^−2^ are shown in Fig. 5d, and it can be seen that the AAEMFC with the A-GDL has lower electrical resistance and mass transfer resistance compared with those of the AAEMFC with the GDL (Sunrise Power). This result also indicates that adjusting the hydrophilicity of the cathode GDL is important in order to improve the performance.

**Fig. 6:**
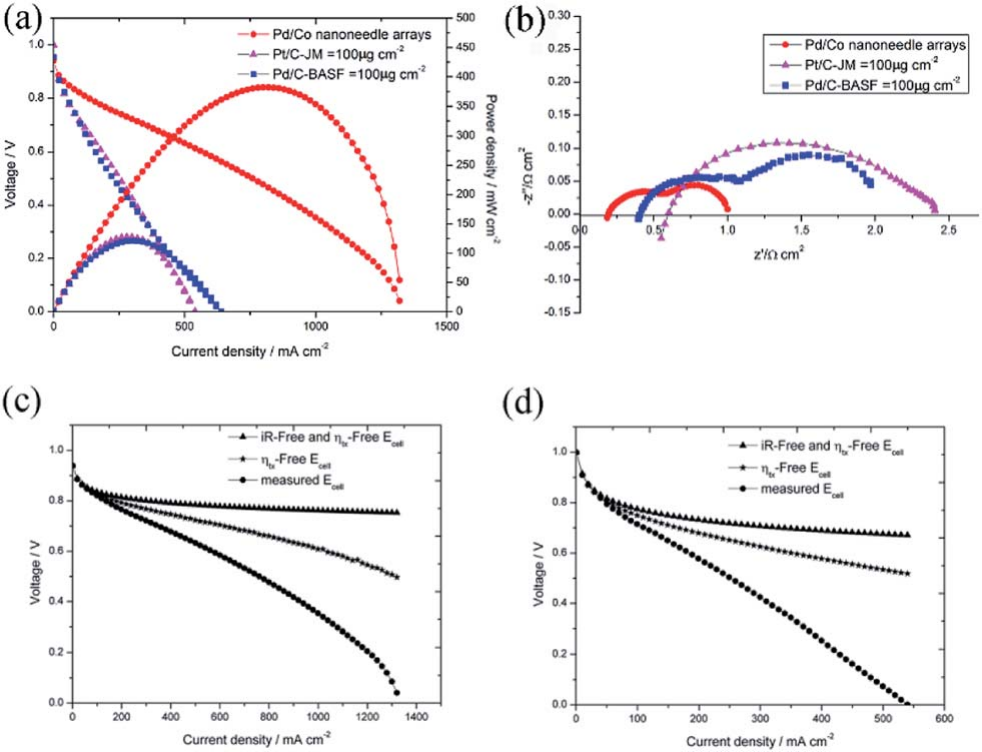
(a) Comparison of the Pd/Co nanoneedle array cathode, which has a Pd loading of 33.5 μg cm^−2^, with a commercial Johnson Matthey 70% Pt/C catalyst cathode (with a Pt loading of 100 μg cm^−2^) and a commercial BASF 20% Pd/C catalyst cathode (with a Pd loading of 100 μg cm^−2^); (b) EIS comparison of the Pd/Co nanoneedle array cathode with the commercial Johnson Matthey 70% Pt/C catalyst cathode and commercial BASF 20% Pd/C catalyst cathode; (c) the ohmic losses and the mass transport losses of the Pd/Co nanoneedle array cathode; (d) the ohmic losses and the mass transport losses of the conventional cathode.

To confirm the superiority of the novel Pd/Co nanoneedle array cathode, the performance of the AAEMFC with the Pd/Co nanoneedle array cathode was compared with that of a conventional cathode electrode. An AAEMFC with a conventional cathode electrode was fabricated using a Johnson Matthey 70% Pt/C electrocatalyst with a Pt loading of 100 μg cm^−2^ and a BASF 20% Pd/C electrocatalyst with a Pd loading of 100 μg cm^−2^. The polarization and power density curves of the Pd/Co nanoneedle arrays, Pd/C cathode electrode and conventional Pt/C cathode electrode are shown in Fig. 6a. Despite the catalyst loading of the novel Pd/Co nanoneedle arrays cathode being 2 times lower than that of the conventional cathode and using Pd as the cathode catalyst, which has a lower activity compared with that of Pt, the AAEMFC with the novel Pd/Co nanoneedle array cathode displays a much higher performance compared to the AAEMFC with the conventional Pt/C and Pd/C cathodes. The maximum power density of the novel Pd/Co nanoneedle array cathode is two times higher than that of the conventional Pt/C and Pd/C cathodes and the maximum current density is 144% higher than that of the conventional Pt/C and Pd/C cathodes. This means that the Pd electrocatalyst on Co nanoneedle arrays fabricated by our method is able to tremendously reduce the total catalyst loading and achieve an excellent performance compared with the conventional Pt/C and Pd/C cathodes. In AAEMFCs, the electrochemical reaction takes place at the three-phase boundaries in the catalyst layer, in which the active catalyst must be simultaneously connected by electrons, reactants and hydroxide ion transfer channels. The conventional catalyst layer is randomly constructed using a catalyst powder and ionomer, where the ionomer is a hydroxide ion conductor, not an electron conductor. Despite the hydroxide ion transfer resistance being significantly decreased by adding an ionomer, some of the catalyst is completely covered by ionomer and is thus not accessible to reactants or electron conduction paths, which decreases the utilization efficiency of the catalyst.^16^ The Pd/Co nanoneedle array catalyst layer has an ordered structure and the Pd catalyst only disperses on the outside of the Co nanoneedle arrays, which are in close contact with the AAEM and gas diffusion layer. Besides this, the pores of the Co nanoneedle arrays are interconnected and open to the surface. These two factors lead to the high utilization efficiency of the Pd catalyst in the Co nanoneedle arrays. Fig. S4† shows the *ex situ* catalytic activity results of the Pd/Co nanoneedles and Pt/C catalyst. From the figure, the *E*_1/2_ value of the Pd/Co nanoneedles (0.792 V *vs.* RHE) is lower than that of the Pt/C catalyst (0.840 *vs.* RHE), which means that the catalytic activity of the Pd/Co nanoneedles is inferior to that of the Pt/C catalyst. However, this is very different from the fuel cell test results, which means that the oriented three-dimensional architecture can significantly improve the catalyst utilization efficiency.

Furthermore, as mentioned above, the thickness of the novel Pd/Co nanoneedle arrays coated on the AAEM is only about 300 nm (Fig. 3b), whereas in contrast, the thickness of the conventional catalyst layer is about 10 μm. Theoretically, the ohmic resistance can be defined as *R* = *ρl*/*S*, where *ρ* is the resistivity of OH^−^ in the catalyst layer, *S* is the cross sectional area of the catalyst layer and *l* is the thickness of the catalyst layer, and the diffusion flux of O_2_ can be written as *J* = *Dc*/*l*, where *D* is the diffusion coefficient of O_2_ in the catalyst layer, *c* is the concentration gradient of O_2_ in the catalyst layer and *l* is the thickness of the catalyst layer. With an increase in *l*, the ohmic resistance of the catalyst layer is higher and the diffusion flux of O_2_ is lower. The thinner the catalyst layer is, the lower the mass transport and ohmic resistance of the catalyst layer is. Additionally, the ohmic losses and the mass-transport losses were quantified to understand the various voltage losses from the cell and the results are shown in Fig. 6c and d, respectively. The results are summarized in Table 1. From the table, the ohmic and mass transport losses from the Pd/Co nanoneedle array cathode are only 40.7% and 26.1% of the values of the losses from the conventional electrode at 200 mA cm^−2^, respectively. At 500 mA cm^−2^, the ohmic and mass transport losses from the Pd/Co nanoneedle array cathode are only 42.3% and 19% of the values of the losses from the conventional electrode, respectively. This means that the Pd/Co nanoneedle array cathode shows much lower ohmic and mass-transport loss values compared with those of the conventional electrode. The results were confirmed by the electrochemical impedance spectra shown in Fig. 6b. The electrochemical impedance spectroscopy (EIS) data recorded at 100 mA cm^−2^ for the Pd/Co nanoneedle arrays show a lower ohmic and electrochemical polarization resistance. The resistance of the Pd/Co nanoneedle arrays as a whole is much smaller than that of the conventional Pt/C catalyst layer. Thus, the Pd/Co nanoneedle array electrode offers a promising MEA structure for use in AAEMFC applications because of its structural advantages.

**Table 1:** A summary of the ohmic losses and mass transport losses of the two electrodes

Material	Thickness	200 mA cm^−2^		500 mA cm^−2^	
		Ohmic loss	Mass transport loss	Ohmic loss	Mass transport loss
Pd/Co nanoneedle array electrode	300 nm	21.4 mV	26.5 mV	61.1 mV	90.2 mV
Conventional electrode	10 μm	52.6 mV	101.4 mV	144.4 mV	473.8 mV

## Conclusion

In summary, we have demonstrated a facile approach to construct a novel cathode catalyst layer with an oriented three-dimensional architecture using Co nanoneedle arrays. The thickness of a Pd/Co nanoneedle array catalyst layer coated on an AAEM is only about 300 nm. This novel ordered Pd/Co nanoneedle array cathode has an excellent cell performance with a two times enhancement in the maximum power density compared with that of traditional Pt/C and Pd/C cathodes. This is attributed to the unique three-dimensional nanoneedle array structure, which increases the utilization efficiency of the catalyst and decreases the ohmic resistance and mass transport resistance of the catalyst layer. The results in this work provide convincing evidence that ordered 3D nanostructures are promising supports for the application of fuel cells and other energy devices.

## Conflicts of interest

There are no conflicts to declare.

## Supplementary Material

RA-008-C7RA13677C-s001
