# Supplementary material for: 3D Pd/Co core–shell nanoneedle arrays as a high-performance cathode catalyst layer for AAEMFCs
Source: RSC Adv. 2018 Apr 5;8(23):12887–93. doi: 10.1039/c7ra13677c (PMC9079337; doi:10.1039/c7ra13677c)
Supplement: RA-008-C7RA13677C-s001 [file RA-008-C7RA13677C-s001.pdf]

Electronic Supplementary Material (ESI) for RSC Advances.
This journal is © The Royal Society of Chemistry 2018

Supporting Information

3D Pd/Co core-shell nanoneedle arrays as high performance cathode catalyst layer for AAEMFCs

Jia Jia[a,b], Hongmei Yu[a,*], Xueqiang Gao[a,b], Jun Chi[a,b], Yachao Zeng[a,b], Bowen Qin[a,b], Dewei Yao[a,b], Wei Song[a], Zhigang Shao [a,*] and BaolianYi[a]

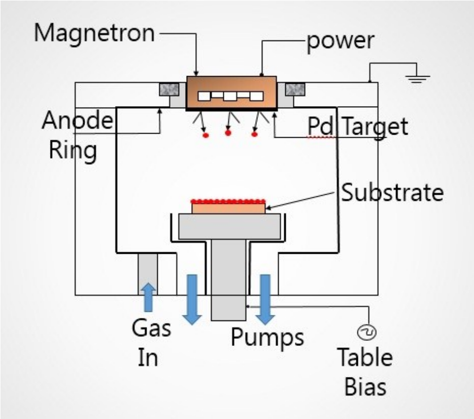

Fig. S1 The setup arrangement of sputtering deposition.

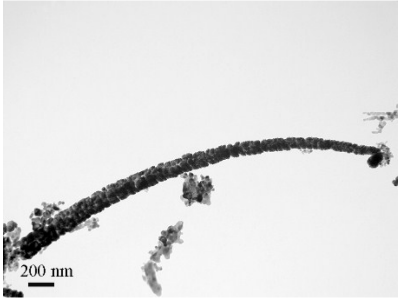

Fig. S2 low magnification TEM image of Pd/Co nanoneedle.

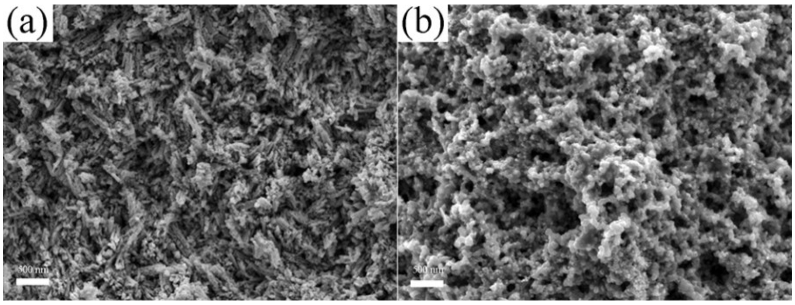

Fig. S3 The SEM images of the prepared catalyst layers coated on the membrane: (a) Pd/Co nanoneedle arrays, (b) conventional Pt/C catalyst layer.

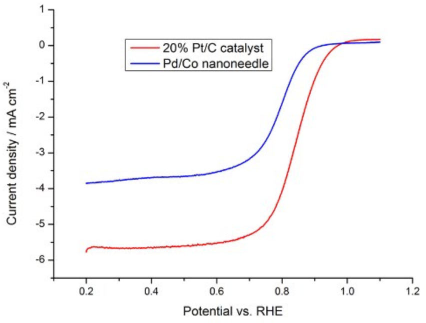

Fig. S4 ORR polarization curves for Pd/Co nanoneedle and 20% Pt/C catalyst. The measurements were performed in 1.0 M O_2-saturated KOH solution. Scan rate: 10 mV s^{-1}. Rotational speed: 1600 rpm.
